# In Silico Comparison of Three Different Beam Arrangements for Intensity-Modulated Proton Therapy for Postoperative Whole Pelvic Irradiation of Prostate Cancer

**DOI:** 10.3390/cancers16152702

**Published:** 2024-07-30

**Authors:** Emile Gogineni, Hao Chen, Ian K. Cruickshank, Andrew Koempel, Aarush Gogineni, Heng Li, Curtiland Deville

**Affiliations:** 1 Department of Radiation Oncology, The Ohio State University Wexner Medical Center, Columbus, OH 43210, USA; andrew.koempel@osumc.edu (A.K.); aarush.gogineni@gmail.com (A.G.); 2 Department of Radiation Oncology and Molecular Radiation Sciences, Johns Hopkins University School of Medicine, Baltimore, MD 21205, USA; hchen142@jhmi.edu (H.C.); ian.cruickshank@bison.howard.edu (I.K.C.J.); hengli@jhmi.edu (H.L.); cdeville@jhmi.edu (C.D.J.)

**Keywords:** prostate cancer, radiation therapy, whole pelvis, pelvic, proton, beam arrangement, intensity-modulated proton therapy, IMPT, pencil beam, scanning beam, dosimetry, dosimetric

## Abstract

**Simple Summary:**

Proton therapy has been shown to provide dosimetric benefits in comparison with IMRT when treating prostate cancer with whole pelvis radiation; however, the optimal proton beam arrangement has yet to be established. Twenty-three post-prostatectomy patients were planned using three different beam arrangements: two-field (opposed laterals), three-field (opposed laterals inferiorly matched to a posterior–anterior beam superiorly), and four-field (opposed laterals inferiorly matched to two posterior oblique beams superiorly) arrangements. CTV coverages were similar for all plans, while the four-field plan provided the lowest doses to several metrics for bladder, bowel, sigmoid, rectum, femoral head, bone, penile bulb, and skin. The data presented herein may help inform the future delivery of whole pelvis IMPT for prostate cancer.

**Abstract:**

**Background and purpose:** Proton therapy has been shown to provide dosimetric benefits in comparison with IMRT when treating prostate cancer with whole pelvis radiation; however, the optimal proton beam arrangement has yet to be established. The aim of this study was to evaluate three different intensity-modulated proton therapy (IMPT) beam arrangements when treating the prostate bed and pelvis in the postoperative setting. **Materials and Methods:** Twenty-three post-prostatectomy patients were planned using three different beam arrangements: two-field (IMPT_2_B) (opposed laterals), three-field (IMPT_3_B) (opposed laterals inferiorly matched to a posterior–anterior beam superiorly), and four-field (IMPT_4_B) (opposed laterals inferiorly matched to two posterior oblique beams superiorly) arrangements. The prescription was 50 Gy radiobiological equivalent (GyE) to the pelvis and 70 GyE to the prostate bed. Comparisons were made using paired two-sided Wilcoxon signed-rank tests. **Results:** CTV coverages were met for all IMPT plans, with 99% of CTVs receiving ≥ 100% of prescription doses. All organ at risk (OAR) objectives were met with IMPT_3_B and IMPT_4_B plans, while several rectum objectives were exceeded by IMPT_2_B plans. IMPT_4_B provided the lowest doses to OARs for the majority of analyzed outcomes, with significantly lower doses than IMPT_2_B +/− IMPT_3_B for bladder V30–V50 and mean dose; bowel V15–V45 and mean dose; sigmoid maximum dose; rectum V40–V72.1, maximum dose, and mean dose; femoral head V37–40 and maximum dose; bone V40 and mean dose; penile bulb mean dose; and skin maximum dose. **Conclusion:** This study is the first to compare proton beam arrangements when treating the prostate bed and pelvis. four-field plans provided better sparing of the bladder, bowel, and rectum than 2- and three-field plans. The data presented herein may help inform the future delivery of whole pelvis IMPT for prostate cancer.

## 1. Introduction

Prostate cancer is the most common malignancy diagnosed in men in the United States and the second most common globally [1,2]. The estimated incidence of prostate cancer in 2024 in the United States is 299,010, causing an estimated 35,250 deaths [3]. Genetic, environmental, and social factors all play potential roles in the development of prostate cancer, with incidence and mortality increasingly proportionally with advancing age [2,4]. It is characterized by a heterogenous cell population, leading to variability in the disease’s natural history and prognosis [5,6]. Epithelial–mesenchymal plasticity has been hypothesized to play a role in prostate cancer’s capability to metastasize and develop resistance to treatment [6].

Treatment options for localized prostate cancer include radical prostatectomy (RP) and radiation therapy (RT) with or without androgen deprivation therapy (ADT). Data from prospective studies comparing RP and RT have shown equal rates of control and survival, with RP resulting in worse incontinence and impotence, and RT leading to worse bowel and rectal irritation [7,8,9]. RP remains the most commonly employed treatment for prostate cancer, with biochemical failure occurring in the majority of patients with adverse pathologic features [10].

Results from multiple randomized prospective trials have proven the biochemical control benefit of postoperative radiation after RP [11,12,13,14,15,16,17,18]. Radiation Therapy Oncology Group 0534, which investigated the benefit of ADT and pelvic irradiation in patients receiving salvage RT, suggests that the addition of whole pelvis RT may provide an additional benefit over RT to the prostate bed alone [19].

Intensity-modulated photon RT (IMRT) is the most widely used radiation technique in the treatment of prostate cancer. Proton therapy has the potential to spare normal tissue in comparison with IMRT due to its rapid dose fall off beyond the target [20]. The dosimetric benefits provided by proton therapy may be more impactful when treating the pelvic lymph nodes due to the large volumes receiving radiation. While prospective data proving benefits are currently lacking, protons have the potential to decrease toxicity, improve quality of life, and reduce rates of second malignancy after treatment.

Intensity-modulated proton therapy (IMPT) using two opposed lateral beams has been shown to reduce the dose to the bladder, bowel, and rectum in comparison to IMRT when treating intact prostate and pelvic nodal fields while providing adequate clinical target volume (CTV) coverage in a dosimetric analysis by Whitaker et al., in which a simultaneous integrated boost was utilized to treat the nodal volume to 45 Gy and the prostate to 67.5 Gy, all in 25 fractions [21]. A single institution, retrospective matched comparison by Santos et al. found that proton therapy to the prostate bed only was superior at reducing the volumes of bladder and rectum receiving 10% to 40% of the dose compared to IMRT [22]. IMPT has also been shown to reduce the dose to organs at risk (OARs) when treating the whole pelvis postoperatively [23].

While IMPT provides dosimetric benefits in comparison with IMRT, the optimal proton beam arrangement has yet to be established when treating with whole pelvis RT. The aim of this study was to evaluate dosimetric differences between three different beam arrangements when treating the prostate bed and pelvic lymph nodes with IMPT in the postoperative setting.

## 2. Materials and Methods

After obtaining approval from our institutional review board, we identified 23 patients with prostate cancer who had undergone initial RP and were then treated with IMPT in the postoperative setting between July 2020 and August 2020. Patients with gross lymphadenopathy and/or distant metastases seen on imaging and those who had received previous irradiation were excluded from analysis.

### 2.1. Simulation

Patients were simulated supine with a full bladder and an empty rectum with a rectal balloon in place. Magnetic resonance imaging (MRI) simulation was obtained immediately following computed tomography (CT) simulation.

### 2.2. Volume Delineation

All CTV and OAR contours were delineated by the attending radiation oncologist in the RayStation treatment planning system (RaySearch Laboratories, Stockholm, Sweden). The CTVs included the pelvic lymph nodes (CTV1) and the postoperative prostate bed (CTV2), delineated according to the Radiation Therapy Oncology Group 0534 protocol [19] and the 2021 NRG Oncology Consensus Pelvic Lymph Node Atlas [24]. CTV1 was combined with CTV2 to create CTV_50.

Planning target volumes (PTVs) were created from CTV expansions according to our institutional protocol. These PTVs were created for evaluation purposes only, as has been described in previous studies, and were not used in planning [25,26,27]. Thus, expansions from CTV to PTV were not identical to the margins used for robust planning. PTV1 was generated using a 5 mm expansion in all directions from CTV1. PTV2 was generated using a 6 mm cranial and caudal expansion from CTV2, and a 5 mm radial expansion from CTV2. PTV_70_Eval was created using a 5 mm lateral margin from PTV_70 to account for range uncertainty, including relative bone thickness change due to prostate interfraction motion. PTV1 was combined with PTV2 to create PTV_50.

Contoured OARs included the bladder, bowel cavity, sigmoid, rectum, femoral heads, penile bulb, bone, and skin. The OAR ‘Rectum (in-field)’ included the portion of the rectum 10 mm superior and inferior to the CTV, while the OAR ‘Rectum (anatomic)’ included the entire anatomic rectum. An additional OAR ‘Bladderless_CTV’ was delineated, which included the entire bladder excluding any overlapping CTV. The OAR ‘Bone’ was generated as a surrogate for bone marrow dose, which was limited to bones 10 mm superior and inferior to the CTV.

### 2.3. Treatment Planning

CTV_50 was prescribed a dose of 50 Gy radiobiological equivalent (GyE) over 25 fractions, which was followed by an additional 10 fraction 20 GyE cone down to CTV2 = CTV_70 to a total of 70 GyE over 35 fractions. A relative biological effectiveness (RBE) dose of 1.1 was used for proton equivalent doses.

**Figure 1:**
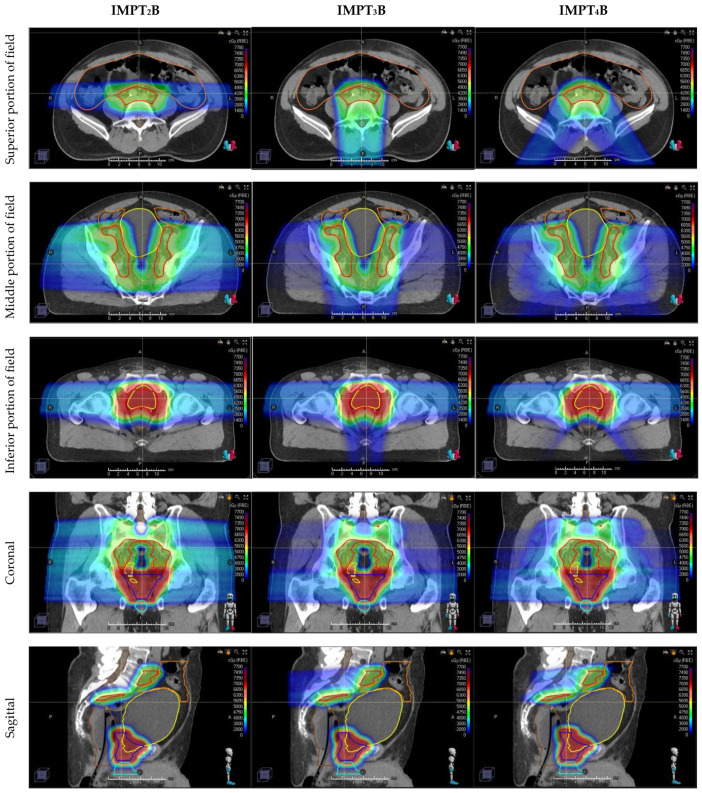
Representative dose-color-wash images in axial (**top three rows**), coronal (**4th row**), and sagittal (**bottom row**) comparing three different proton beam arrangements, with the dose increasing as the color ranges from blue to green to red. Axial images are divided into superior (**1st row**), middle (**2nd row**), and inferior (**3rd row**) portions of the target. Compared plans include 2-field: IMPT_2_B (**left**), 3-field: IMPT_3_B (**middle**), and 4-field: IMPT_4_B (**right**) plans.

Three sets of IMPT plans were prepared for each patient with predefined CTV and OAR dose-volume histogram (DVH) objectives as outlined in Table 1. These plans were created using 3 different beam arrangements: 2-field (IMPT_2_B) (opposed laterals), 3-field (IMPT_3_B) (opposed laterals inferiorly matched to a posterior–anterior [PA] beam superiorly), and 4-field (IMPT_4_B) (opposed laterals inferiorly matched to 2 posterior oblique [PO] beams superiorly) arrangments. PO beams were delivered 25–35 degrees off from the PA direction. For IMPT_3_B and IMPT_4_B, the PA and PO beams were arranged to cover the superior portion of the target until the base of the prostate and then fade out using a gradient match. The lateral beams began superiorly where there was no bowel lateral to the CTV and covered the entirety of the remaining inferior portion of the target optimized with a single-field optimization (SFO) technique. The dose in the overlapped regions for lateral beams and PA/PO beams was gradient matched in the superior and inferior directions. Figure 1 depicts an example of the dose-color-wash for each plan.

Plans differed in how the dose was delivered to the superior portion of the field, in which beams were arranged with 2 opposed lateral beams for IMPT_2_B, a single posterior–anterior beam for IMPT_3_B, and 2 posterior oblique beams for IMPT_4_B. These beam arrangements covered the target until the base of the prostate, then faded out using a gradient match to the inferior portion of the field, which was covered by opposed laterals for all 3 plans.

**Figure 2:**
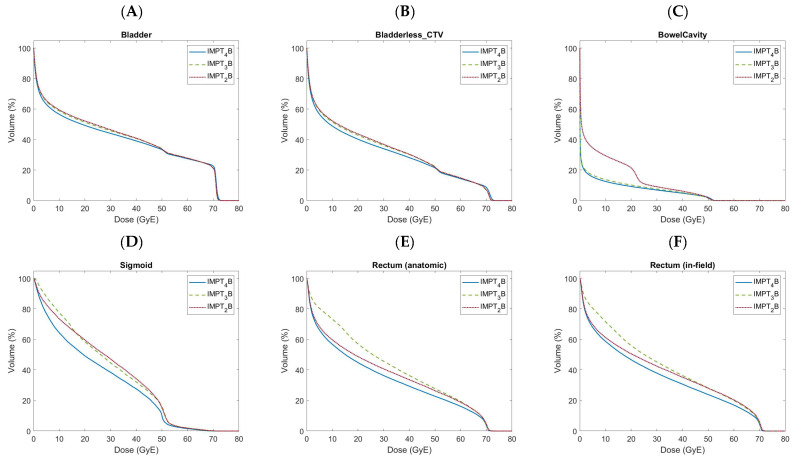

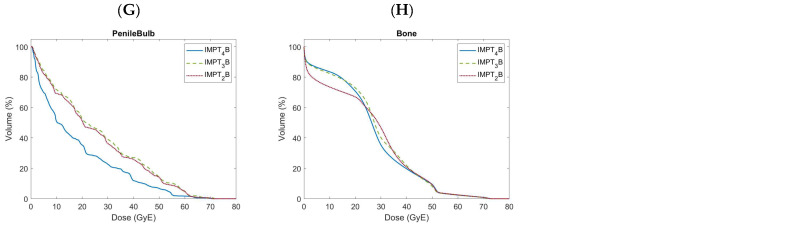
Doses to organs at risk shown over the entire dose-volume histogram comparing 2-(IMPT_2_B), 3-(IMPT_3_B), and 4-field (IMPT_4_B) proton beam arrangements, including the bladder (**A**), bladderless_CTV (**B**), bowel (**C**), sigmoid (**D**), anatomic rectum (**E**), in-field rectum (**F**), penile bulb (**G**), and bone (**H**).

As shown in the axial images (1st row), the opposed lateral beams range directly through the bowel in the superior portion of the field, which correlates with higher doses to the bowel with the IMPT_2_B plans seen in Table 1 and Figure 2C. Figures from the inferior portion of the field (3rd row) depict the increased conformality of the dose deposited posterior to the prostate with IMPT_4_B plans, correlating with the lower rectal doses shown in Table 1 and Figure 2E,F.

Plans were generated by multiple proton-specialist dosimetrists using the RayStation treatment planning system. Proton plans were created with pencil beam scanning (IMPT) using inverse optimization with robustness with a setup uncertainty of 3 mm and a range uncertainty of 3.5% using fast graphics processing units (GPU) Monte Carlo optimization. The goal was to cover PTV_70_Eval with 95% of the prescription isodose in both lateral directions to evaluate for the robustness for range uncertainty, daily skin surface uncertainty, and the daily related motion to bone anatomy.

### 2.4. Statistical Analysis

The volumetric percentage of CTVs and OARs along the entire DVH were evaluated. Comparative maximum and mean OAR doses were also assessed. The paired 2-sided Wilcoxon signed-rank test was used to compare the plans with *p* < 0.05 considered statistical significance. Statistical analyses were performed using Matlab R2024a (MathWorks, Natick, MA, USA) and Microsoft Office LTSC Professional Plus 2021 Excel (Microsoft, Redmond, WA, USA).

## 3. Results

Table 1 provides comparative values for target coverage and the doses to OARs. Comparative DVH’s for OARs are shown in Figure 2A–H.

### 3.1. Target Coverage

CTV_50 and CTV_70 coverages were met for all IMPT plans, with 99% of CTVs receiving ≥ 100% of prescription doses. CTV coverages were numerically highest with IMPT_4_B plans for all objectives except the volume of CTV_70 receiving 70.0 GyE, which was highest with IMPT_2_B plans (not statistically significant). There were no significant differences in CTV coverage between plans.

### 3.2. Organ at Risk Objectives

The volume of in-field rectum receiving 60.0 GyE exceeded objectives for IMPT_2_B plans. The maximum dose to both the anatomic and the in-field rectum exceeded objectives for IMPT_2_B plans. All OAR objectives were met for IMPT_3_B and IMPT_4_B plans.

### 3.3. Genitourinary Organs at Risk

Volumes of the irradiated bladder (Figure 2A) and bladderless_CTV (Figure 2B) were numerically lowest with IMPT_4_B plans for all objectives except for volumes of the bladder and bladderless_CTV receiving 70.0 GyE and the maximum dose to bladderless_CTV, which were lowest with IMPT_3_B plans. Volumes of the bladder and bladderless_CTV receiving 30.0 and 45.0 GyE were significantly lower with IMPT_4_B plans than with both IMPT_2_B and IMPT_3_B plans, while volumes receiving 50.0 GyE were significantly lower with IMPT_4_B plans than with IMPT_3_B plans. Volumes of the bladder and bladderless_CTV receiving 70.0 GyE and maximum doses were significantly higher with IMPT_4_B plans than those with both IMPT_2_B and IMPT_3_B plans. Mean doses to the bladder and bladderless_CTV were numerically lowest with IMPT_4_B plans, with significantly lower mean doses than those with IMPT_2_B and IMPT_3_B plans.

### 3.4. Gastrointestinal Organs at Risk

Volumes of the irradiated bowel cavity (Figure 2C) were numerically lowest with IMPT_4_B plans for all objectives < 45.0 GyE and lowest with IMPT_3_B plans for objectives ≥ 45.0 GyE. Volumes of the bowel cavity receiving 15.0 and 30.0 GyE were significantly lower with IMPT_3_B plans than with IMPT_2_B plans and significantly lower with IMPT_4_B plans than with both IMPT_2_B and IMPT_3_B plans. Volumes of the bowel cavity receiving 40.0 and 45.0 GyE were significantly higher with IMPT_2_B plans than with both IMPT_3_B plans and IMPT_4_B. The mean dose to the bowel was numerically lowest with IMPT_4_B plans, with a significantly lower mean dose than IMPT_2_B and IMPT_3_B plans. IMPT_3_B plans also had a significantly lower mean dose to the bowel than IMPT_2_B plans.

The maximum dose to the sigmoid (Figure 2D) was numerically lowest with IMPT_4_B plans, with significantly lower maximum doses than IMPT_2_B and IMPT_3_B plans.

Volumes of the irradiated anatomic (Figure 2E) and the in-field rectum (Figure 2F) were numerically lowest with IMPT_4_B plans for all doses. Volumes of the anatomic and the in-field rectum receiving 40.0, 50.0, 60.0, and 70.0 GyE were significantly lower with IMPT_4_B plans than with both IMPT_2_B and IMPT_3_B plans. Volumes of the anatomic and the in-field rectum receiving 70.0 GyE were also significantly lower with IMPT_3_B plans than with IMPT_2_B plans. Volumes of the anatomic and the in-field rectum receiving 72.1 GyE were significantly lower with IMPT_4_B plans than IMPT_2_B plans. Maximum doses to the anatomic and the in-field rectum were significantly lower with IMPT_4_B plans than with both IMPT_2_B and IMPT_3_B plans, while IMPT_3_B plans also had significantly lower maximum doses than IMPT_2_B plans. Mean doses to the anatomic and the in-field rectum were numerically lowest with IMPT_4_B plans, with significantly lower mean doses than those with IMPT_2_B and IMPT_3_B plans. IMPT_3_B plans also had significantly higher mean doses to the anatomic and the in-field rectum than IMPT_2_B plans.

### 3.5. Bony Organs at Risk

Volumes of the irradiated left and right femoral heads were numerically lowest with IMPT_4_B plans for all doses. Volumes of each femoral head receiving 37.0 and 40.0 GyE were significantly lower with IMPT_4_B plans than with IMPT_2_B plans. The maximum dose to each femoral head was significantly lower with IMPT_4_B plans than with both IMPT_2_B and IMPT_3_B plans.

The volume of bone (Figure 2H) receiving 10.0 GyE was numerically lowest with IMPT_2_B plans and was significantly lower than that with both IMPT_3_B and IMPT_4_B plans. The volume of bone receiving 40.0 GyE was numerically lowest with IMPT_4_B plans and was significantly lower than that with IMPT_3_B plans. The mean dose to bone was numerically lowest with IMPT_2_B plans, with a significantly lower mean dose than with IMPT_3_B and IMPT_4_B plans. IMPT_4_B plans also had a significantly lower mean dose to bone than IMPT_3_B plans.

### 3.6. Other Organs at Risk

The mean dose to the penile bulb (Figure 2G) was numerically lowest with IMPT_4_B plans, with a significantly lower mean dose than with IMPT_2_B and IMPT_3_B plans.

The maximum dose to skin was numerically lowest with IMPT_4_B plans and was significantly lower than with both IMPT_2_B and IMPT_3_B plans.

## 4. Discussion

This is the first study to our knowledge comparing proton beam arrangements when treating the prostate bed and elective pelvic lymph nodes in the postoperative setting. We found that the four-field IMPT beam arrangement showed the greatest reductions in low-to-intermediate doses to OARs, particularly in the bowel and rectum, compared to the two- and three-field arrangement. IMPT_4_B also provided the lowest mean dose to the bladder, bowel, rectum, and penile bulb while maintaining similar or better target coverage.

### 4.1. Beam Arrangements

There has been limited data published to date evaluating differences between varying proton beam arrangements when treating the pelvic lymph nodes with IMPT. Butala et al. suggested that the treatment of pelvic lymph nodes using a three-field approach with a posterior field matched inferiorly with opposed lateral beams to treat the prostate improves OAR sparing in comparison with a plan solely utilizing opposed lateral beams to treat both the prostate and the whole pelvis. They did not address the use of a four-field beam arrangement for proton irradiation using PO beams to treat the pelvis, as is carried out in the study presented herein.

Regarding the treatment of intact prostate cancer and the prostate target volume, Tang et al. compared three different arrangements of proton beams, including equally weighted bilateral fields, a single straight anterior field, and two equally weighted anterior oblique (AO) fields. They found that AO fields decreased the dose to the anterior rectal wall and femoral heads in comparison with lateral fields [28]. Underwood et al. compared four treatment plans, including photon IMRT, passively scattered opposed lateral proton beams, passively scattered AO proton beams, and AO IMPT [29]. They found that plans utilizing AO beam arrangements typically did not meet both tumor and OAR constraints, often resulting in substantial hotspots within the rectum. Both of these studies focused on the treatment of the prostate alone and did not evaluate patients receiving whole pelvis radiation therapy (WPRT), nor did they include the three- and four-field beam arrangements used in our analysis.

A study from the University of Florida by Chera et al. compared photon IMRT to a four-field proton plan to the intact prostate, seminal vesicles, and pelvic lymph nodes [30]. They used opposed lateral fields to treat the prostate and four fields to treat the pelvis, including two lateral and two PO beams. Despite the use of double-scattered proton beams with uniform intensities, they did find a reduction in the dose to OARs using this arrangement in comparison with IMRT. However, they did not utilize modern proton scanning beam technology and did not compare this beam arrangement to others, such as the IMPT_2_B, IMPT_3_B, and IMPT_4_B comparison described in our cohort.

### 4.2. Correlation between Dosimetric Outcomes and Toxicity

There are no prospective proton therapy studies to date assessing whether these dosimetric differences between beam arrangements translate to reductions in toxicity and improved quality of life. However, gastrointestinal (GI) toxicity has been shown to correlate with the volume of the irradiated rectum. Kuban et al. suggested that the volumes of the rectum receiving low doses of radiation may be even more significant in predicting rectal morbidity [31,32,33,34]. Our study shows a reduction in the volume of the rectum receiving doses of 40, 50, 60, and 70 GyE with the IMPT_4_B arrangement (see Table 1 and Figure 2E,F). It also demonstrates a reduction in the volume of the bowel receiving 45 GyE in the IMPT_3_B and IMPT_4_B plans and the lowest volume of the bowel receiving 15 GyE in the IMPT_4_B plan (see Table 1 and Figure 2C), which have both been shown to be predictive of grade ≥ 3 acute GI toxicity [35].

Bryant et al. showed that grade 3 genitourinary (GU) toxicity was significantly associated with bladder V30 on univariate (hazard ratio [HR]: 0.4) and multivariate analysis (HR: 0.5) [36]. We found that bladder and bladderless_CTV V30 were lowest with the IMPT_4_B plan, which was significantly lower than that of IMPT_2_B and IMPT_3_B plans (see Table 1 and Figure 2A,B).

### 4.3. Robustness

Movement-induced dose reduction occurs significantly more with the proton irradiation of the prostate using opposed lateral beams when compared to photon irradiation [37,38,39]. This is due to variations in femur rotation, the thickness of subcutaneous adipose tissue, and changes in bladder and bowel filling. Yoon et al. showed that a lateral shift of 6 mm decreased coverage by 9% for a proton plan when treating with opposed lateral beams, as opposed to only 1% for a photon IMRT plan [38]. These sensitivities of coverage for proton irradiation can be mitigated by the use of IMPT with appropriate planning strategies and minimizing motion with serial cone-beam CT imaging and immobilization devices such as a rectal balloon [40,41].

The effect of beam arrangement on plan robustness is multifactorial. For example, the majority of patients have a slight indent at midline in the posterior lumbar region. Thus, a small lateral shift could have a greater effect on the PA beam used in the IMPT_3_B plan than on the PO beams used in the IMPT_4_B plan, given that the PA beam traverses directly through this indent. On the other hand, the larger number of beams used in the IMPT_4_B plan increases the time required to deliver each fraction and could affect the robustness of the plan due to the potential that the patient could move. Given the complexity of this concept and the difficulty accounting for each of these variables, we did not perform a formal comparison of robustness between beam arrangements.

### 4.4. Limitations

This study is limited by the number of patients included. While 23 patients may not provide a robust cohort to make strong conclusions when analyzing clinical outcomes in a retrospective study, it is significantly larger than most dosimetric studies, which typically do not include more than 10 patients [21,23,30,42,43,44,45]. All patients underwent simulation and treatment planning with rectal balloons in place. Correspondingly, conclusions made from our study should not be extrapolated to settings in which rectal balloons are not used.

Another limitation of this study was the lack of proof that the dosimetric outcomes found will correlate directly with quality of life and toxicity. However, further optimization of beam arrangement and delivery allowing increasing plan conformality has the potential to maximize the benefits provided by the unique physical properties of proton irradiation. This may lead to clearer clinical benefits over time. Our group is conducting analyses using normal tissue complication probability models in order to assess whether the dosimetric findings from our study may predict differences in toxicity and quality of life. Ultimately, clinical data are necessary to understand differences in patient outcomes.

## 5. Conclusions

This study is the first to provide a dosimetric comparison of proton beam arrangements when treating the prostate bed and pelvic lymph nodes. Mean and low-to-intermediate doses to organs at risk were lower with the four-field plan than with the two- and three-field plans, particularly for the bladder, bowel, and rectum. The data presented herein may help inform the future delivery of proton therapy for prostate cancer in the postoperative setting.

## Figures and Tables

**Table 1:** Comparative dosimetric outcomes for IMPT 2-, 3-, and 4-field plans. Mean and standard deviation values are provided as percentages or cubic centimeters (as denoted in the column labeled “Clinical Goal”) for volumetric endpoints and as GyE for mean and maximum dose endpoints.

Target/OAR	Clinical Goal	IMPT_2_B		IMPT_3_B		IMPT_4_B		*p*-Value		
		Mean	St-Dev	Mean	St-Dev	Mean	St-Dev	_2_B vs _3_B	_2_B vs _4_B	_3_B vs _4_B
**CTV_50**	V 49.0 GyE > 98.0%	100	0.1	100	0.0	100	0.0	0.348	0.215	0.162
	V 50.0 GyE > 98.0%	99.6	0.6	99.6	0.6	99.8	0.2	0.864	0.117	0.136
**CTV_70**	V 68.6 GyE > 99.0%	100	0.0	100	0.0	100	0.0	N/A	N/A	N/A
	V 70.0 GyE > 99.0%	99.8	0.4	99.8	0.5	99.7	0.5	0.519	0.282	0.375
**Bladder**	**V 30.0 GyE < 50.0%**	46.5	9.6	45.8	10.6	43.7	9.2	0.337	**<0.001**	**0.004**
	**V 45.0 GyE < 40.0%**	37.7	9.5	37.7	10.0	36.3	9.1	0.887	**<0.001**	**0.001**
	**V 50.0 GyE < 35.0%**	33.8	9.6	34.1	9.8	33.3	9.4	0.274	0.129	**0.002**
	V 65.0 GyE < 20.0%	25.5	9.1	25.5	9.2	25.4	9.0	0.973	0.933	0.927
	**V 70.0 GyE < 15.0%**	21.3	8.4	21.2	8.4	22.7	8.6	0.123	**<0.001**	**<0.001**
	**Mean Dose (GyE)**	31.3	6.4	31.0	6.8	29.9	6.4	0.453	**<0.001**	**0.003**
**Bladderless_CTV**	**V 30.0 GyE < 50.0%**	36.9	7.6	36.2	8.2	33.7	6.5	0.364	**<0.001**	**0.003**
	**V 45.0 GyE < 40.0%**	26.5	6.0	26.6	6.5	24.9	5.4	0.829	**<0.001**	**0.001**
	**V 50.0 GyE < 35.0%**	21.9	5.5	22.3	5.8	21.4	5.1	0.227	0.145	**0.003**
	V 65.0 GyE < 20.0%	12.0	3.8	12.0	3.9	12.0	3.7	1.000	0.986	0.979
	**V 70.0 GyE < 15.0%**	7.1	2.4	6.9	2.5	8.7	2.9	0.123	**<0.001**	**<0.001**
	**D 0.03 cm^3^ < 73.5 GyE**	72.5	0.6	72.4	0.5	73.0	0.3	**0.036**	**0.002**	**<0.001**
	**Mean Dose (GyE)**	24.1	4.4	23.9	4.8	22.6	4.1	0.494	**<0.001**	**0.002**
**Bowel Cavity**	**V 15.0 GyE < 830.0 cm^3^**	530.0	202.7	219.8	92.8	200.0	83.7	**<0.001**	**<0.001**	**<0.001**
	**V 30.0 GyE < 300.0 cm^3^**	170.1	71.7	140.8	61.1	131.4	57.9	**<0.001**	**<0.001**	**0.002**
	**V 40.0 GyE < 30.0%**	6.2	4.1	5.2	3.6	4.9	2.9	**<0.001**	**<0.001**	0.086
	**V 45.0 GyE < 195.0 cm^3^**	81.2	37.6	68.8	32.2	70.5	33.9	**<0.001**	**<0.001**	0.298
	V 55.0 GyE < 20.0 cm^3^	0.7	2.0	0.7	2.1	0.8	2.2	0.446	0.262	0.510
	V 60.0 GyE < 5.0 cm^3^	0.4	1.3	0.4	1.4	0.5	1.5	0.445	0.224	0.489
	**Mean Dose (GyE)**	8.8	3.3	4.9	3.1	4.6	2.6	**<0.001**	**<0.001**	**0.013**
**Sigmoid**	**D 0.03 cm^3^ < 66.0 GyE**	59.0	7.1	58.8	6.9	56.7	7.0	0.599	**<0.001**	**<0.001**
**Rectum** **(anatomic)**	**V 40.0 GyE < 40.0%**	33.7	8.2	34.7	9.7	28.9	6.8	0.160	**<0.001**	**<0.001**
	**V 50.0 GyE < 30.0%**	26.8	7.3	26.9	8.4	22.3	5.8	0.822	**<0.001**	**<0.001**
	**V 60.0 GyE < 20.0%**	19.5	6.0	19.0	6.5	15.8	4.6	0.209	**<0.001**	**<0.001**
	**V 70.0 GyE < 10.0%**	5.5	2.2	4.7	2.0	4.0	1.9	**<0.001**	**<0.001**	**0.009**
	**V 72.1 GyE < 0.5 cm^3^**	0.1	0.2	0.1	0.2	0.0	0.0	0.102	**0.038**	0.266
	**D 0.03 cm^3^ < 72.1 GyE**	72.1	0.6	71.6	0.6	71.2	0.3	**<0.001**	**<0.001**	**0.005**
	**Mean Dose (GyE)**	27.3	5.2	29.4	6.0	24.6	4.5	**0.001**	**<0.001**	**<0.001**
**Rectum** **(in-field)**	**V 40.0 GyE < 40.0%**	34.7	8.8	35.8	10.4	30.2	7.4	0.162	**<0.001**	**<0.001**
	**V 50.0 GyE < 30.0%**	27.7	7.8	27.8	9.0	23.3	6.3	0.825	**<0.001**	**<0.001**
	**V 60.0 GyE < 20.0%**	20.2	6.4	19.7	7.0	16.6	4.9	0.204	**<0.001**	**<0.001**
	**V 70.0 GyE < 10.0%**	5.7	2.2	4.8	2.0	4.1	1.9	**0.001**	**<0.001**	**0.009**
	**V 72.1 GyE < 0.5 cm^3^**	0.1	0.2	0.1	0.2	0.0	0.0	0.114	**0.039**	0.270
	**D 0.03 cm^3^ < 72.1 GyE**	72.1	0.6	71.6	0.6	71.2	0.3	**<0.001**	**<0.001**	**0.005**
	**Mean Dose (GyE)**	28.1	5.5	30.2	6.3	25.6	4.8	**0.001**	**<0.001**	**<0.001**
**Left Femoral Head**	**V 37.0 GyE < 50.0%**	0.9	1.3	0.1	0.3	0.1	0.1	**0.004**	**0.002**	0.470
	**V 40.0 GyE < 40.0%**	0.2	0.3	0.0	0.1	0.0	0.1	**0.016**	**0.003**	0.788
	V 50.0 GyE < 10.0%	0.0	0.0	0.0	0.0	0.0	0.0	N/A	N/A	N/A
	**D 0.03 cm^3^ < 53.0 GyE**	40.2	3.2	35.6	3.4	34.2	3.9	**<0.001**	**<0.001**	**0.015**
**Right Femoral Head**	**V 37.0 GyE < 50.0%**	1.0	1.3	0.2	0.7	0.0	0.1	**0.009**	**0.002**	0.312
	**V 40.0 GyE < 40.0%**	0.1	0.2	0.1	0.3	0.0	0.0	0.546	**0.017**	0.361
	V 50.0 GyE < 10.0%	0.0	0.0	0.0	0.0	0.0	0.0	N/A	N/A	N/A
	**D 0.03 cm^3^ < 53.0 GyE**	40.3	3.3	36.1	3.5	34.4	4.1	**<0.001**	**<0.001**	**0.006**
**Bone**	**V 10.0 GyE < 90.0%**	73.5	5.1	82.4	5.0	83.4	3.4	**<0.001**	**<0.001**	**0.027**
	**V 40.0 GyE < 37.0%**	20.8	4.9	21.8	3.9	19.6	3.3	0.163	0.060	**0.000**
	**Mean Dose (GyE)**	26.0	2.5	27.5	2.1	26.6	1.9	**0.001**	**0.038**	**0.001**
**Penile Bulb**	**Mean < 52.5 GyE**	23.6	6.2	23.7	7.3	22.0	6.6	0.848	**0.003**	**<0.001**
**Skin**	**D 0.03 cm^3^ < 56.0 GyE**	36.4	2.4	36.8	1.5	31.0	1.7	0.652	**<0.001**	**<0.001**

Abbreviations: IMPT: intensity-modulated proton therapy, OAR: organ at risk, _2_B: 2-field plan, _3_B: 3-field plan, _4_B: 4-field plan, St-Dev: standard deviation, CTV: clinical target volume, GyE: radiobiological Gy equivalent.

## Data Availability

Research data are stored in an institutional repository and will be shared upon request to the corresponding author.

## Abbreviations

RP	Radical prostatectomy
RT	Radiation therapy
ADT	Androgen deprivation therapy
IMRT	Intensity-modulated photon radiation therapy
IMPT	Intensity-modulated proton therapy
CTV	Clinical target volume
OAR	Organ at risk
MRI	Magnetic resonance imaging
CT	Computed tomography
PTV	Planning target volume
GyE	Gy radiobiologic equivalent
RBE	Relative biological effectiveness
DVH	Dose-volume histogram
IMPT_2_B	two-field IMPT beam arrangement using opposed laterals
IMPT_3_B	three-field IMPT beam arrangement using opposed laterals inferiorly matched to a Posterior–anterior beam superiorly
PA	Posterior–anterior
IMPT_4_B	four-field IMPT beam arrangement using opposed laterals inferiorly matched to 2 Posterior oblique beams superiorly
PO	Posterior oblique
SFO	Single-field optimization
GPU	Graphics processing units
AO	Anterior oblique
WPRT	Whole pelvis radiation therapy
GI	Gastrointestinal
GU	Genitourinary
HR	Hazard ratio
